# Does the Regulatory Environment for E-Cigarettes Influence the Effectiveness of E-Cigarettes for Smoking Cessation?: Longitudinal Findings From the ITC Four Country Survey

**DOI:** 10.1093/ntr/ntx056

**Published:** 2017-04-05

**Authors:** Hua-Hie Yong, Sara C Hitchman, K Michael Cummings, Ron Borland, Shannon M L Gravely, Ann McNeill, Geoffrey T Fong

**Affiliations:** 1 Cancer Council Victoria, Melbourne, Australia;; 2 Department of Addictions, Institute of Psychiatry, Psychology, and Neuroscience, King’s College London, London, UK;; 3 UK Centre for Tobacco and Alcohol Studies (UKCTAS), Nottingham, UK;; 4 Department of Psychiatry and Behavioral Sciences, Medical University of South Carolina, Charleston, SC;; 5 Department of Psychology, University of Waterloo, Waterloo, Canada;; 6 School of Public Health and Health Systems, University of Waterloo, Waterloo, Canada;; 7 Ontario Institute for Cancer Research, Toronto,Canada

## Abstract

**Introduction:**

To date, no studies have explored how different regulatory environments may influence the effectiveness of electronic cigarettes (ECs) as a smoking cessation aid.

**Objective:**

This study compares the real-world effectiveness of adult smokers using ECs for quitting compared with quitting unassisted or quitting with nicotine replacement therapy (NRT) and/or prescription medications in two countries with restrictive policies towards ECs (ie, Canada and Australia) versus two countries with less restrictive policies (ie, United States and United Kingdom).

**Methods:**

Data were drawn from the International Tobacco Control Four Country surveys, from the United States and Canada (2 waves, *n* = 318 and 380, respectively), the United Kingdom (3 waves, *n* = 439) and Australia (4 waves, *n* = 662), collected 2010–2014. Smokers at baseline wave who reported making a quit attempt at follow-up were included. The primary outcome was self-reported abstinence for at least 30 days regardless of smoking status at follow-up assessment. Data across waves were combined and analyzed using generalized estimating equations.

**Results:**

Compared to unassisted quitting (ie, no medications or ECs), smokers who used ECs for quitting from countries with less restrictive EC policy environments were more likely (OR = 1.95, 95%CI = 1.19–3.20, *p* < .01), whereas smokers who used ECs for quitting from countries with more restrictive EC policies were less likely (OR = 0.36, 95%CI = 0.18–0.72, *p* < .01), to report sustained abstinence for at least 30 days.

**Conclusions:**

Use of ECs in the real world during a quit attempt appears only effective for sustaining smoking abstinence in a less restrictive EC environment suggesting that the benefits of ECs for smoking cessation are likely highly dependent on the regulatory environment.

**Implications:**

What this study adds: This is the first study to examine the impact of regulatory environment for ECs on their real-world effectiveness for smoking cessation. This study shows that in a less restrictive EC regulatory environment, use of ECs during a quit attempt facilitates, but in a more restrictive environment, it inhibits, short-term sustained abstinence. The findings underscore the need for careful consideration on how best to regulate this emerging product so that EC benefits for smoking cessation are maximized and its risks to public health are minimized.

## Introduction

Globally, the reported prevalence of electronic cigarette (EC) use has increased in recent years.^1^ The increased use of e-cigarettes has fueled debate about whether such products will benefit or harm public health.^2^ Those who oppose ECs are concerned about possible adverse impacts of EC use on nonsmokers, especially young people who would otherwise not smoke or use nicotine-containing products.^3–5^ There is also concern about the real benefits of e-cigarettes because of dual use by smokers and unknown health risks from long-term use.^6,7^ Proponents of EC use have emphasized the potential benefits to help smokers who are unable or unwilling to quit, to stop smoking.^8^ Current evidence suggests that ECs are less harmful than conventional cigarettes, and in fact ECs have shown to improve lung function among smokers in a recent randomized controlled trial (RCT)^9^ although ECs may not completely be without any risk particular among those with pre-existing health conditions such as cardiovascular disease or cancer.^10,11^

ECs may represent a unique and new paradigm for affecting the cessation of conventional cigarettes, although currently the effectiveness of ECs for helping smokers to quit is uncertain. Recent meta-analytic reviews of RCTs have shown that the odds of quitting conventional cigarettes were twice as high in those using ECs with nicotine compared with those using ECs without nicotine.^12,13^ However, a meta-analysis of real-world observational studies came to the opposite conclusion, suggesting that the odds of quitting were lower for smokers who used ECs compared to those who did not use ECs.^14^ A recent study by Kalkhoran and Glantz^15^ reviewed findings from clinical trials and observational studies and concluded that smokers who used or had used ECs were less likely to have stopped smoking than those who had not used ECs. This review, however, did not consider three aspects of ECs and their usage that have been found to be important in the impact of ECs on quitting. First, it did not consider whether or not smokers were using ECs to quit; a recent study has found that smokers who used ECs to quit in their last quit attempt (LQA) were significantly more likely to quit^16^ and so combining these smokers with those who were using ECs for other purposes unrelated to quitting would dilute the positive impact of ECs on quitting. Second, the review did not take into account frequency and duration of the use of e-cigarettes; studies have shown that greater frequency and duration (ie, daily use for at least a month) is associated with a greater likelihood of quitting.^17,18^ Third, the review treated all ECs the same, despite the many dimensions on which these products vary, including dimensions related to potential nicotine delivery; a recent study has shown that tank systems were associated with greater likelihood of quitting, relative to other types of e-cigarettes (eg, cigalikes, disposables).^13^

It is presently unclear whether the use of ECs is associated with greater cigarette abstinence than current practice (ie, use of nicotine replacement therapy [NRT], prescription medications [PM] or behavioral therapy) as the majority of the studies to date have not included a comparison to either currently approved quitting methods (eg, NRT, PM) or no EC use. A recent real-world cross-sectional study found that smokers who used ECs were more likely to report abstinence than those who used a licensed NRT product bought over the counter or no aid to cessation.^16^

The present study examines the possible impact of the restrictiveness of the regulatory environment for ECs on quitting among smokers. Availability and access to nicotine-based ECs are likely to be affected by the regulatory environment for ECs.^19^ In countries like Australia and Canada where the retail sales of nicotine containing ECs are effectively banned, it would be more difficult for smokers to obtain ECs with nicotine, and hence, they are likely to have less opportunity to experiment with different devices to find ones that best suited to them in terms of nicotine delivery. By contrast, in countries like the United States and the United Kingdom, where ECs are more widely available and nicotine-containing ECs are more accessible, users of ECs have greater opportunities to vape and experiment with different nicotine-containing devices, which may improve their likelihood of being able to quit smoking. Recent evidence indicates that smokers’ awareness and use of ECs, especially ones with nicotine, are lower in Australia and Canada than in the United Kingdom and the United States consistent with the difference in EC regulatory environment.^1,20^

Using longitudinal data from the International Tobacco Control (ITC) Four Country Survey, this study aimed to compare the relative effectiveness of using ECs as compared with other methods to quit smoking among adult smokers in two countries with more restrictive regulatory environments for e-cigarettes (Canada and Australia) versus two countries with less restrictive regulatory environments (United States and United Kingdom). We hypothesized that the quit rate would be higher among smokers who used ECs for quitting compared to those using no help but no different from an approved method, and that EC effectiveness for quitting would be higher in a less restrictive, than in a more restrictive, EC environment.

## Methods

### Sample

Study participants came from the ITC Four Country Survey, a longitudinal cohort survey of a nationally representative sample of adult smokers (aged ≥18) from the United States, the United Kingdom, Australia, and Canada, followed up approximately annually. Those lost to the study were replenished using the same sampling procedure as initial recruitment. Details of the ITC conceptual model and methodologies have been reported elsewhere.^21,22^ For this study, the analytic sample consisted of only those who provided data for at least one wave-to-wave transition, defined as smoking at one wave (baseline) and at the next wave (follow-up) reported having made at least one quit attempt since the baseline wave. To ensure sufficient sample size, data from several waves of the ITC surveys were combined and analyzed (see Table 1). The study period spanned between 2010 and 2014 across the four countries where data were collected using a mix of telephone interviews and web surveys.

**Table 1.** Survey Waves, Wave-to-Wave Transitions and Sample Sizes for the Study Sample^a^ in Each Country

Country	Survey wave (year) conducted	Wave-to-wave transitions	Total combined across waves, N/n^b^
United Kingdom	Wave 8 (2010)	Wave 8–9; Wave 9–10	439/487
	Wave 9 (2013)		
	Wave 10 (2014)		
United States	Wave 8 (2010)	Wave 8–9	318/318
	Wave 9 (2013)		
Australia	Wave 8 (2010)	Wave 8–8.5; Wave 8.5–9; Wave 9–10	662/855
	Wave 8.5 (2011)		
	Wave 9 (2013)		
	Wave 10 (2014)		
Canada	Wave 8 (2010)	Wave 8–9	380/380
	Wave 9 (2013)		

^a^The study sample consisted of those in each wave-to-wave transition who reported smoking at baseline wave and had made at least one quit attempt by the follow-up wave.

^b^ *N* = number of unique individuals; *n* = number of observations.

### Measures

#### Outcome Variable (30-Day Sustained Abstinence)

The primary outcome was self-reported abstinence for at least 30 days regardless of smoking status at follow-up. This was derived from responses to several questions asked of smokers at follow-up assessment. At follow-up, participants still smoking were asked whether they had made a quit attempt since last survey. In addition, those quit at follow-up were coded as having made a quit attempt. All quit attempters were asked when their most recent quit attempt started (in days). Anyone quit for less than 30 days was excluded as their quit length could not be determined. Those who relapsed to smoking following a quit attempt were also asked when their most recent quit attempt ended to compute the duration of their most recent quit attempt, which was then used to derive the outcome.

#### Predictor Variables (Help Used at Last Quit Attempt)

Smokers who made a quit attempt were asked what forms of help, including an opportunity to specify any other help, they might have received for their last/current quit attempts as well as whether they used any (NRT, such as nicotine patch, gum, and lozenge) or (PM, such as varenicline or bupropion) for their last/current quit attempts. Use of ECs was not specifically asked until the 2014 (Wave 10) survey (United Kingdom and Australia only) using the question: “Did you use an e-cigarette as part of your last quit attempt?” For earlier waves, use of ECs for LQA was derived from two open-ended questions on what other NRT and other help they used for last quit attempt. For the purpose of analysis, help at LQA was defined as follows: no help was defined as not using any ECs, NRT, or PM for last quit attempts; ECs, NRT, and PM were each defined as exclusive use of each method for last quit attempts; combination help was defined as any combination of the aforementioned methods for last quit attempts (eg, ECs plus NRT; ECs plus NRT plus PM). Each of the help categories above could include behavioral support (defined as any advice or information about quitting smoking from a telephone/Quitline service, an internet website about quitting smoking, or a local stop-smoking service [clinics/specialists]). Because what constituted behavioral support was heterogeneous across countries, a decision was made not to use it as a criterion for defining help except for the sensitivity analysis. In the 2014 survey, additional questions were added to assess, among those who reported using ECs for their last quit attempts, the frequency of EC use at the start of their quit attempts (every day, some days, or not at all) and the product type used (disposables, replaceable pre-filled cartridges, or liquid filled tank systems). Those who reported ever used an EC were asked whether their current/last EC contained nicotine.

#### Moderator Variable

Country was used as a proxy for the regulatory environment for ECs. The key regulatory differences between countries are presented in Table 2. For the purpose of this study, United Kingdom and the United States were coded as a less restrictive, while Australia and Canada were coded as a more restrictive, EC regulatory environment.

**Table 2.** EC Policies in the United Kingdom, United States, Australia, and Canada During the Study Period, 2010–2014

Less restrictive EC environments		More restrictive EC environments	
United Kingdom	United States	Australia	Canada
• ECs (both nicotine and nicotine free products) sold as consumer products	• No federal laws on the sale and marketing of ECs	• No specific federal laws but existing laws may apply in some circumstances	• Federal ban on sale, advertising, and import of ECs unless products licensed by Health Canada; None licensed so far
• Manufacturers can apply for medicinal license with MHRA (Medicines Healthcare Products Regulatory Agency)—no licensed products on market in United Kingdom during 2010–2014	• ECs only subject to regulation by FDA if therapeutic claims are made	• Retail sale, import and personal possession/use of nicotine ECs is unlawful without a permit or a doctor’s prescription	• Nicotine-free ECs with no health claims can be sold legally or imported
• No national rules on age limit or use in smoke-free public areas	• No federal laws on use in smoke-free public places or age limit but some states and localities have banned the use of ECs in public places and have established age limits on sales	• Nicotine-free ECs are legal to import and sell but several states have banned them because they resemble tobacco products	• No federal laws on sale to minors
• Advertising and promotion allowed, some oversight through Advertising Standards Authority	• No federal taxes on ECs, although some states have enacted taxes on ECs	• No federal laws on use in smoke-free public areas but in 2014, some states have banned ECs from smoke-free public places	• No federal laws on use in smoke-free public places although use in indoor public places are banned in some provinces and cities
• Any product licensed as medicines subject to advertising rules of the Medicines and Healthcare Products Regulatory Agency		• Certain promotions may be considered in breach of the Tobacco Advertising Prohibition Act 1992	
• 2014 European Union Tobacco Products Directive to be implemented in May 2016 will regulate all ECs with nicotine not licensed as medicines			

EC = electronic cigarettes; FDA = Food and Drug Administration.

#### Covariates

Socio-demographics including age, sex, ethnicity (white vs. non-white in the United States, United Kingdom, and Canada; English-speaking vs. non-English speaking in Australia), income and education assessed at prior wave (baseline) along with smoking-related variables such as baseline Heaviness of Smoking Index^23^ to assess nicotine dependence, baseline quitting interest, baseline any quit attempt within last 6 months, number of quit attempts made, recency of last quit attempt, and methodological variables such as survey wave, survey mode at each wave (phone vs. web interview), year recruited into the study, and inter-wave intervals (time between waves).

### Data Analysis

All data analyses were conducted using Stata version 14. To account for the different number of waves of data available for analyses between countries (four waves in Australia, three in the United Kingdom and two each in the United States and Canada) and also the correlated nature of the data due to repeated measurement, generalized estimating equations (GEE) models were employed to assess the association between different forms of help at LQA and 30-day sustained abstinence while controlling for covariates. Marginal quit rate by form of help was computed for each individual country and similar regulatory environment using the post-estimation command “margins” in Stata to obtain the adjusted predictions at the means. For modelling the binary outcome (30-day sustained abstinence vs. not), parameter estimation was conducted using the logit link function with robust variance and an unstructured correlation structure for our working correlation matrix. To test for significant moderating effects of regulatory environment, a dummy variable indicating more restrictive versus less restrictive EC regulatory environment was derived and used to create an interaction term ‘regulation by help at LQA’ for inclusion in the models. Statistical significance was set at alpha = 0.05.

## Results

### Sample Characteristics

Table 3 presents the demographic and smoking-related characteristics of the analytic sample. Compared to respondents from countries with a more restrictive EC regulatory environment, a greater proportion of those from countries with a less restrictive EC regulatory environment were older, from low income background, of lower nicotine dependence at baseline, had no quit interest at baseline, had not made a recent quit attempt at baseline, and the target quit date was earlier in the inter-survey interval. In addition, based on a 2014 survey where data on EC use patterns were available in Australia and the United Kingdom, 70.2% of the United Kingdom smokers who made a quit attempt using ECs reported using ECs on a daily basis (compared to 58.9% in Australia), 45.5% reported using a tank system (compared to 35.9%) and 75.3% reported using ones that contain nicotine as compared to 41.0% of their Australian counterparts (data not shown).

**Table 3.** Sample Characteristics

Variables	Less restrictive EC regulatory environment			More restrictive EC regulatory environment			Significant difference by regulatory environment
	United Kingdom *N* = 439	United States *N* = 318	Combined *N* = 757	Australia *N* = 662	Canada *N* = 380	Combined *N* = 1042	
Demographics							
Age groups (%)							
18–29	5.3	2.5	4.2	4.3	4.7	4.5	*p* **=** .013
30–39	12.9	6.9	10.6	13.6	11.8	13.0	
40–54	36.6	32.1	34.8	39.4	39.4	39.4	
≥55	45.2	58.1	50.4	42.8	44.1	43.2	
Sex-Female	54.2	56.9	55.3	53.9	55.9	54.6	*p* = .752
Baseline income (%)							
Low	58.5	35.2	49.3	28.9	18.1	25.6	*p* < .001
Moderate	21.2	29.9	24.6	27.0	34.9	29.4	
High	12.7	28.6	19.0	38.2	39.4	38.6	
No information	7.6	6.3	7.1	5.9	7.6	6.5	
Baseline education(%)							
Low	47.2	41.8	45.1	51.9	33.9	46.3	*p* = .051
Moderate	26.9	38.1	31.3	29.6	43.6	33.9	
High	24.6	20.1	22.9	18.5	22.3	19.6	
Ethnicity (%)							
white	95.3	86.8	91.9	91.5	93.4	92.1	*p* = .091
Smoking related variables							
Baseline HSI							
*M* (*SD*)	2.2(1.5)	2.4(1.5)	2.3(1.5)	2.6(1.6)	2.6(1.6)	2.6(1.5)	*p* < .001
Baseline quit interest							
Yes(%)	41.5	41.2	41.4	50.6	48.3	49.9	*p* < .001
Baseline quit attempts within last 6 months							
Yes(%)	33.9	41.5	36.9	46.3	33.9	42.4	*p* = .012
Number of attempts (%)							
1	56.7	39.3	49.8	53	43.6	50.1	*p* = .816
2	25.1	22.3	24	24.7	24.9	24.7	
≥3	15.2	38.4	24.4	20.3	31.5	23.8	
Don’t know	3.1	0	1.9	2	0	1.4	
Quit recency (%)							
1 to <6 m	49.7	55.0	51.8	67.1	42.3	59.4	*p* < .001
6 to <12 m	27.5	21.4	25.1	25.2	24.2	24.9	
12 m or more	20.3	23.6	21.6	6.5	33.6	14.9	
Don’t Know	2.5	0	1.5	1.2	0	0.8	
Survey mode (%)							
Web–web	45.8	22	36.4	37.2	34.1	36.2	*p* = .074
Phone–web	19.3	20.4	19.8	15.2	21.3	17.1	
Web–phone	3.5	5	4.1	7	5.3	6.5	
Phone–phone	31.4	52.5	39.8	40.7	39.4	40.3	
Interwave intervals							
<2 y	20.5	0	12.4	100	0	69.2	*p* < .001
2 to <3 y	79.5	27	58.8	0	18.9	5.8	
3 y or more	0	73	28.8	0	81.1	25	
Year (Wave) recruited into the study (*n*)							
2002 (Wave 1)	76	39	115	164	77	241	*p* <.001
2003 (Wave 2)	11	10	21	23	23	46	
2004 (Wave 3)	40	19	59	56	25	81	
2005 (Wave 4)	45	30	75	46	31	77	
2006 (Wave 5)	71	43	114	107	48	155	
2007 (Wave 6)	88	66	154	152	83	235	
2008 (Wave 7)	56	44	100	34	43	77	
2010 (Wave 8)	0	67	67	93	51	144	
2011 (Wave 8.5)	0	0	0	93	0	93	
2013 (Wave 9)	100	0	100	88	0	88	

HSI = Heaviness of Smoking Index. Data presented in the table based on Waves 8, 8.5, 9, and 10 for Australia, Waves 8, 9, and 10 for the United Kingdom, and Waves 8 and 9 for both the United States and Canada.

### Predicting Sustained one-month abstinence

GEE analyses revealed that there was an overall significant interaction between forms of help at LQA and regulatory environment on 1-month sustained abstinence (*p* < .001; differential effect found only for ECs but not the other forms of help) and thus regulation-stratified analyses were conducted. Table 4 presents the results stratified by regulatory environment (see Supplementary Table S1 for individual country results). In the less restrictive countries—the United States and the United Kingdom (see Table 4), about 58% of the smokers who reported quitting without using any NRT, PM, or ECs reported 30-day sustained abstinence as compared to 73% who used ECs to quit, 70% who quit using NRT, 74% who used PM, 68% who used a combination of help and 68% of those who responded “Don’t Know” (DK). GEE results indicated that compared to the no help group, those who quit using ECs were significantly more likely to sustain abstinence for at least 30 days (odds ratio [OR] = 1.95, 95% confidence interval [CI] = 1.19–3.20, *p* < .01) and similarly those who quit using PM were significantly more likely to sustain abstinence for at least 30 days (OR = 2.07, 95% CI = 1.14–3.77, *p* < .05). Those who quit using NRT or a combination help, or responded DK were also more likely to report 30-day sustained abstinence but this did not reach statistical significance compared to no help (OR = 1.64, 1.51, and 1.54, respectively). When compared to the group using ECs for LQA, those using NRT and PM and also those who used a combination of help showed similar likelihood of being able to sustain at least 30 days abstinence (see Table 4).

**Table 4.** Generalized Estimating Equations (GEE) Results Showing the Effectiveness of ECs for Sustaining at least 30-Day Abstinence (as Compared to ‘no help’ Group and as Compared to Various Approved Methods) by EC Regulatory Environment

Variable	Less restrictive EC policies *N* = 757; *n* = 805				More restrictive EC policies *N* = 1042; *n* = 1235			
	*n*	%quit	AOR	95%CI	*n*	%quit	AOR	95%CI
Using ‘No help’ as the comparison group:								
Help at LQA								
no meds/no ecig	308	58.4	ref		555	56.2	ref	
Ecig only	145	73.2	1.95	(1.19–3.20)**	50	31.5	0.36	(0.18–0.72)**
NRT only	144	69.7	1.64	(0.99–2.71)	286	59.6	1.15	(0.83–1.61)
PM only	100	74.4	2.07	(1.14–3.77)*	222	68.5	1.69	(1.17–2.46)**
Combination help	98	67.9	1.51	(0.89–2.56)	114	56.4	1.01	(0.62–1.64)
Don’t Know/No info	10	68.4	1.54	(0.32–7.55)	8	45.1	0.64	(0.16–2.62)
Using ‘electronic cigarette’ as the comparison group:								
Help at LQA								
Ecig only	145	73.2	ref		50	31.5	ref	
no meds/no ecig	308	58.4	0.51	(0.31–0.84)**	555	56.2	2.78	(1.39–5.55)**
NRT only	144	69.7	0.84	(0.47–1.50)	286	59.6	3.20	(1.56–6.59)**
PM only	100	74.4	1.06	(0.53–2.11)	222	68.5	4.71	(2.25–9.86)***
Combination help	98	67.9	0.78	(0.43–1.39)	114	56.4	2.80	(1.25–6.26)*
Don’t Know/No info	10	68.4	0.79	(0.16–3.85)	8	45.1	1.78	(0.38–8.27)

AOR = adjusted odds ratios; LQA = last quit attempt; *N* = number of individuals; *n* = number of observations; NRT = nicotine replacement therapy; PM = stop-smoking prescription meds (varenicline or buproprion). Model adjusted for survey wave, age groups, sex, country, ethnicity, baseline educ, baseline income, baseline HSI, baseline quit intention, baseline recent quit attempt, number of quit attempts, quit recency, survey mode, interwave interval and wave of recruitment; % quit estimates adjusted for covariates in the GEE model.

*p < .05; **p < .01; ***p < .001.

In the more restrictive countries—Australia and Canada (see Table 4), the 30-day sustained abstinence rate was 56% for those who quit without help but by contrast, the quit rate for those using ECs for their LQA was considerably lower at 32% with the quit rate being higher only for PM (69%) and no difference for the group quitting using NRT (60%) or a combination of help (56%), or who responded DK (45%). GEE results indicated that those who quit using ECs were significantly less likely to sustain abstinence for at least 30 days compared with those with no help (OR = 0.36, 95% CI = 0.18–0.72, *p* < .01) whereas those who quit using PM were more likely to do so (OR = 1.69, 95% CI = 1.17–2.46, *p* < .01) with no difference for the group quitting using either NRT (OR = 1.15) or a combination help (OR = 1.01), or who responded DK (OR = 0.64). Compared to those using ECs for their LQA, the likelihood of sustaining abstinence for at least 30 days was significantly higher for NRT and PM (OR = 3.20, 95% CI = 1.56–6.59, *p* < .01; and OR = 4.71, 95% CI = 2.25–9.86, *p* < .001, respectively) and also the combination help group (OR = 2.80, 95% CI = 1.25–6.26, *p* < .05).

### Sensitivity Analysis

We explored the inclusion of behavioral support as an additional criterion for defining the forms of help categories and the results were similar (see Supplementary Table S2). We also explored data available in the 2014 surveys for the United Kingdom and Australia on EC use frequency and product type at last quit attempts to determine their possible impact on quitting outcome; unfortunately, however, the sample sizes in Australia were too low for meaningful analysis, but fewer used tank systems. In the United Kingdom, frequency of use of ECs was not significantly associated with quit outcome but product type used was positively associated with those using a tank system to quit being more likely to sustain abstinence for at least 30 days compared to disposables/replaceable cartridges (OR = 2.57, 95% CI = 0.83–8.01, *p* = .10) and significantly more likely than no help (OR = 3.11, 95% CI = 1.08–8.93, *p* < .05).

We also explored the association of nicotine content of ECs with outcome using the 2014 survey data. Logistic regression results indicated that the odds of 30-day sustained abstinence were higher among those using nicotine-based ECs than among those using nicotine-free ECs in both the United Kingdom (OR = 3.07, 95% CI = 0.33–27.79) and Australia (OR = 1.32, 95% CI = 0.19–9.07) although this was not statistically significant, owing to small sample sizes.

## Discussion

The results from this study are consistent with the notion that the strictness of the regulatory environment for ECs can affect their real-world effectiveness for quitting. In the United States and the United Kingdom, which have fewer restrictions on marketing and sale of ECs, smokers who used ECs for their last quit attempts were almost twice as likely to quit for at least 30 days compared to those who quit without using ECs or any approved therapy. By contrast, in Australia and Canada, which have more restrictive EC regulatory environments, those who used ECs to quit were significantly less likely to sustain abstinence for 30 days or more compared to those who quit without help. The effects were striking because they were significant and opposite, suggesting that the impact of the regulatory environment can be either facilitative or inhibitory.

The reasons for the facilitative effect of ECs for quitting being found only in countries with a less restrictive environment for ECs could be because quitters from these countries may have better access to products, to more effective products (ie, tanks systems with nicotine solutions) and greater opportunity to access new supplies when needed; or a generally more supportive environment that is actively supportive of EC use. This may result in them having greater opportunities to experiment with different devices until they find a suitable device. Use patterns and product type have been shown in past studies to be important for ECs to be an effective quitting method.^17,18^ Unfortunately data on frequency of use and product type collected only in our 2014 survey were insufficient to adequately test the explanatory hypotheses we proffer here, but the trends in both tank system use and nicotine use are strongly suggestive that they contribute partly to the observed differences. However, product inferiority cannot explain the observed inferiority over no aids in the more restrictive environments. The poorer outcome may be due to restrictions on ready access to products and greater social disapproval, but also could be due to self-selection bias. To succeed in quitting smokers have to use the product consistently for weeks, if not longer, and both poor access to the product and perhaps reluctance to vape in public around potentially hostile others are plausible reasons for thinking poor compliance would be a factor in the high failure rates. In Australia at least, ECs are seen as less socially acceptable.^24^ It is also possible that in low-availability countries, those who chose to use ECs to quit were those who were desperate, having failed to quit using approved methods, and were destined to fail unless ECs provided a quantum increase in effectiveness over approved methods, something which is unlikely.^12^

The finding of approximately equal effectiveness of ECs and NRT for quitting in less restrictive EC policy environment is contrary to that found by Brown et al.^16^ where ECs were shown to be superior to NRT for quitting among a population-based sample of adult smokers in England. One possible reason for the discrepant findings might have to do with how forms of quitting assistance were defined. Unlike Brown et al.,^16^ we did not include behavioral support, or separate NRT over the counter from NRT on prescription in defining our help groups, although reanalysing our data including behavioral support as a criterion did not make any difference in our sensitivity analysis. Another alternative may have been different patterns of use of tank systems and/or non-nicotine products, but it may simply be a chance effect, suggesting ECs, at least the types available at the time of the study, were not a lot more effective than other forms of NRT. The superior outcome of approved therapies such as NRT and PM to ECs in restrictive EC environments both adds to the ecological validity of our findings, and suggests that current practice may be a better option for those who want to quit in restrictive environments than in less restrictive ones, unless they think they can overcome the additional problems related to EC use that occur in the restrictive environments. Given the evidence that PM were superior to no help, irrespective of the EC regulatory environment, the use of PM such as varenicline should be recommended where the smokers are prepared to use them. The advice for NRT is less clear. Although not significant, the odds ratios for NRT compared to no help are similar to those found in meta-analyses of RCTs.^25,26^ Further, as we have previously shown, in studies like this, the effects of help are attenuated the longer the recall period,^27^ and unfortunately we did not have enough cases to limit the period to one short enough to show likely real effects. The problem with existing effective aids is that many smokers are not prepared to use them,^28^ or to use them for long enough to sustain abstinence.^29^ The greater interest in using EC, particularly in England, shows that e-cigarettes are likely leading to extra quitting because they offer a method of help to people who would have otherwise not quit or sought help, even if they led to no additional odds of success on any given attempt.^30^

### Strengths and Limitations

This study has several strengths. First, this study directly assessed the use of ECs for last quit attempts among smokers, removing any confounding with other reasons for the use of ECs, thus allowing a better evaluation of the effectiveness of ECs for quitting relative to approved methods. Second, this study included data from two pairs of countries with divergent policy environments for ECs, thus providing a more definitive evaluation of the impact of EC policy environment on the effectiveness of using ECs for quitting than single country comparisons could, as it reduces the likelihood of conflating other country characteristics with policy environment. Third, while the interactions are meaningful, the absolute levels of effect compared with no treatment need to be viewed with caution as the likely benefit of all forms of help is likely underestimated due to differential memory effect.^27^ The common effect found for approved medicines adds credibility to our argument that the findings with regard to ECs are specific to the policy environment.

Several other limitations warrant some discussion. First, it was not possible to control for whether ECs used for last quit attempts contained nicotine and the type of devices used. Among the minority where this information was available, use of non-nicotine was more common in the more restrictive environment (at least of Australia). However, if these factors contributed to the difference, they would simply reflect mechanisms by which the policy environment had its effects. Second, because data on the use of ECs for LQA were extracted from open-ended responses for earlier waves, it is possible that we might have under-estimated the prevalence of use of ECs for quitting. Third, long-term quit outcome was not evaluated in this study, so we can say nothing about effects on subsequent relapse.

### Policy Implications

The findings from this study suggest that the effectiveness of ECs for smoking cessation may depend on the regulatory environment for ECs. While the use of ECs for quitting has risen rapidly in recent years irrespective of regulatory environment,^31^ the benefits of ECs for smoking cessation may be limited to those who reside in an environment where there are few restrictions on the retail sale and marketing of ECs. If these results are confirmed, it underscores the need for careful consideration of how best to regulate this emerging product. Even if ECs are no more effective than other effective treatments, the higher levels of use as found in the less restrictive environment of the United Kingdom, both here and in other studies,^30^ suggests ECs will have a markedly greater impact on overall cessation rates, overcoming a major limitation with NRT products which are not used much.^28^ Any form of product regulations should maximize overall public health benefits while not preventing or inhibiting innovations that are critical for continued improvement of ECs and other alternative nicotine delivery systems (ANDS).^32^ Whether ECs should be regulated as a medicine, a tobacco product or a consumer product and the impacts it might have on its efficacy as a smoking cessation aid remain somewhat unclear. However, we note that all the ECs used in this study must have been purchased as consumer products as there are no ECs available as therapeutic goods, so clearly consumer products are being used to quit smoking, albeit in some cases substituting vaping for smoking rather than quitting nicotine altogether. The United States, United Kingdom, Canada, Australia and other countries worldwide are moving towards different regulatory approaches and will provide further opportunities to study their impacts on EC effectiveness as a cessation aid.

### Conclusions

In a less restrictive EC regulatory environment, use of ECs during a quit attempt in the real world facilitates, but in a more restrictive environment, where use is lower, it appears to inhibit, short-term sustained smoking abstinence, thus suggesting that the benefits of ECs for smoking cessation may be dependent on the regulatory environment. If so, developing an appropriate regulatory framework for ECs should be a priority so that the benefits of ECs for smoking cessation can be realized, while not neglecting potential risks. Where the regulatory environment supports it, given the popularity of ECs, smokers who are unable or unwilling to quit using current approved methods should be offered the option of quitting with ECs or replacing smoking with ECs for harm reduction purposes.^33^

## Supplementary Material

Supplementary data are available at *Nicotine & Tobacco Research* online.

## Funding

The ITC Four Country Survey during 2010–2014 was supported by multiple grants from the US National Cancer Institute including R01 CA100362, P01 CA138389, and P01 CA138389-06S1. Additional grant support was provided by the Canadian Institutes of Health Research (79551, 115016, and FDN-14877), and National Health and Medical Research Council of Australia (265903, 450110, APP1005922). Work conducted for this article was also partially supported by grants from the US National Cancer Institute (P01 CA200512) and National Health and Medical Research Council of Australia (APP1106451). AM and SCH are members of the UK Centre for Tobacco & Alcohol Studies, a UK Clinical Research Collaboration Public Health Research: Centre of Excellence whose work is supported by funding from the Medical Research Council, British Heart Foundation, Economic and Social Research Council, and the National Institute for Health Research under the auspices of the UK Clinical Research Collaboration (MR/K023195/1). AM and SCH are also funded by Cancer Research UK (C25586/A19540). GTF is supported by a Senior Investigator Award from the Ontario Institute for Cancer Research and a Prevention Scientist Award from the Canadian Cancer Society Research Institute. SMLG is supported by a Canadian Cancer Society Career Development Award in Prevention.

## Declaration of Interests

*KMC has received grant funding from Pfizer, Inc. to study the impact of a hospital based tobacco cessation intervention and also has served as an expert witness in litigation filed against the tobacco industry. All other authors have no conflicts of interest to declare.*

## Supplementary Material

Supplementary Table S1-S2
