# Supplementary material for: Does the Regulatory Environment for E-Cigarettes Influence the Effectiveness of E-Cigarettes for Smoking Cessation?: Longitudinal Findings From the ITC Four Country Survey
Source: Nicotine Tob Res. 2017 Apr 5;19(11):1268–76. doi: 10.1093/ntr/ntx056 (PMC5896424; doi:10.1093/ntr/ntx056)
Supplement: Supplementary Table S1-S2 [file ntx056_suppl_supplementary_table_s1-s2.docx]

**Supplementary Tables**

Supplementary Table S1. Effectiveness of ECs as compared to other quit methods for sustaining at least one-month abstinence: results presented separately by country

UK – GEE models predicting one-month sustained abstinence using data combined across Waves 9 and 10

| Variable | Model 1a: (no meds/ecig as ref)  N=439; n=487 | | | | Model 1b: (ecig as ref)  N=439; n=487 | | | |
| --- | --- | --- | --- | --- | --- | --- | --- | --- |
|  | n | %quit | AOR | 95%CI | n | %quit | AOR | 95%CI |
| Help at LQA |  |  |  |  |  |  |  |  |
| no meds/no ecig | 155 | 63.9 | ref |  | 155 | 63.9 | 0.53 | (0.28-0.99)* |
| ecig only | 117 | 77.1 | 1.90 | (1.01-3.58)* | 117 | 77.1 | ref |  |
| NRT only | 102 | 73.7 | 1.58 | (0.83-3.02) | 102 | 73.7 | 0.83 | (0.41-1.67) |
| PM only | 46 | 83.5 | 2.85 | (0.97-8.35) | 46 | 83.5 | 1.50 | (0.48-4.64) |
| Combination help/DK | 67 | 72.2 | 1.47 | (0.71-3.03) | 67 | 72.2 | 0.77 | (0.38-1.58) |

US – Logistic regression models predicting one-month sustained abstinence using Wave 9 data

| Variable | Model 2a: (no meds/ecig as ref)  N=318 | | | | Model 2b: (ecig as ref)  N=318 | | | |
| --- | --- | --- | --- | --- | --- | --- | --- | --- |
|  | n | %quit | AOR | 95%CI | n | %quit | AOR | 95%CI |
| Help at LQA |  |  |  |  |  |  |  |  |
| no meds/no ecig | 153 | 52.1 | ref |  | 153 | 52.1 | 0.37 | (0.13-1.08) |
| ecig only | 28 | 74.4 | 2.67 | (0.92-7.71) | 28 | 74.4 | ref |  |
| NRT only | 43 | 64.1 | 1.64 | (0.73-3.71) | 43 | 64.1 | 0.61 | (0.18-2.05) |
| PM only | 54 | 68.9 | 2.04 | (0.95-4.36) | 54 | 68.9 | 0.76 | (0.24-2.47) |
| Combination help/DK | 40 | 64.3 | 1.65 | (0.71-3.88) | 40 | 64.3 | 0.62 | (0.18-2.11) |

Australia – GEE models predicting one-month sustained abstinence using data combined across Waves 8.5, 9 and 10

| Variable | Model 3a: (no meds/ecig as ref)  N=662; n=855 | | | | Model 3b: (ecig as ref)  N=662; n=855 | | | |
| --- | --- | --- | --- | --- | --- | --- | --- | --- |
|  | n | %quit | AOR | 95%CI | n | %quit | AOR | 95%CI |
| Help at LQA |  |  |  |  |  |  |  |  |
| no meds/no ecig | 380 | 50.8 | ref |  | 380 | 50.8 | 2.02 | (0.89-4.57) |
| ecig only | 37 | 36.8 | 0.50 | (0.22-1.12) | 37 | 36.8 | ref |  |
| NRT only | 202 | 55.2 | 1.24 | (0.83-1.84) | 202 | 55.2 | 2.50 | (1.07-5.83)* |
| PM only | 151 | 61.3 | 1.68 | (1.07-2.64)* | 151 | 61.3 | 3.39 | (1.41-8.16)** |
| Combination help/DK | 85 | 53.4 | 1.13 | (0.64-2.00) | 85 | 53.4 | 2.29 | (0.89-5.85) |

Canada – Logistic regression models predicting one-month sustained abstinence using Wave 9

| Variable | Model 4a: (no meds/ecig as ref)  N=380 | | | | Model 4b: (ecig as ref)  N=380 | | | |
| --- | --- | --- | --- | --- | --- | --- | --- | --- |
|  | n | %quit | AOR | 95%CI | n | %quit | AOR | 95%CI |
| Help at LQA |  |  |  |  |  |  |  |  |
| no meds/no ecig | 175 | 68.4 | ref |  | 175 | 68.4 | 8.28 | (1.76-38.87)** |
| ecig only | 13 | 20.7 | 0.12 | (0.03-0.57)** | 13 | 20.7 | ref |  |
| NRT only | 84 | 66.0 | 0.90 | (0.48-1.70) | 84 | 66.0 | 7.43 | (1.53-36.13)* |
| PM only | 71 | 76.9 | 1.54 | (0.76-3.12) | 71 | 76.9 | 12.74 | (2.56-63.53)** |
| Combination help/DK | 37 | 57.6 | 0.63 | (0.27-1.48) | 37 | 57.6 | 5.21 | (0.99-27.48) |

Note. N, number of individuals; n, number of observations; AOR, adjusted odds ratios; NRT, nicotine replacement therapy; PM, stop-smoking prescription meds (varenicline or buproprion); LQA, last quit attempt; *p<.05; **p<.01;

Model adjusted for survey wave, age groups, sex, ethnicity, baseline educ, baseline income, baseline HSI, baseline quit intention, baseline recent quit attempt, number of quit attempts, quit recency, survey mode, interwave interval & wave of recruitment; % quit estimates adjusted for covariates in the GEE/logistic regression model;

Supplementary Table S2. GEE results using an alternate definition of ‘no help’ that excludes any use of behavioural support

One-month sustained abstinence (no meds/no ecig/no beh sup as reference)

| Variable | Less restrictive EC policies  N=757; n=805 | | | | More restrictive EC policies N=1042; n=1235 | | | |
| --- | --- | --- | --- | --- | --- | --- | --- | --- |
|  | n | %quit | AOR | 95%CI | n | %quit | AOR | 95%CI |
| Help at LQA |  |  |  |  |  |  |  |  |
| no meds/ecig/behsup | 295 | 58.8 | ref |  | 528 | 55.6 | ref |  |
| ecig only | 141 | 72.6 | 1.86 | (1.13-3.08)* | 47 | 30.7 | 0.35 | (0.17-0.72)** |
| NRT only | 99 | 59.9 | 1.05 | (0.60-1.83) | 260 | 58.5 | 1.12 | (0.79-1.59) |
| PM only | 73 | 74.9 | 2.09 | (1.08-4.07)* | 204 | 67.8 | 1.68 | (1.14-2.48)** |
| Beh support  Combo/DK/No info | 13  184 | 44.7  75.0 | 0.57  2.11 | (0.13-2.47)  (1.32-3.38)** | 27  169 | 65.4  60.3 | 1.51  1.21 | (0.64-3.56)  (0.81-1.81) |

One-month sustained abstinence (ecig as reference)

| Variable | Less restrictive EC policies  N=757; n=805 | | | | More restrictive EC policies  N=1042; n=1235 | | | |
| --- | --- | --- | --- | --- | --- | --- | --- | --- |
|  | n | %quit | AOR | 95%CI | n | %quit | AOR | 95%CI |
| Help at LQA |  |  |  |  |  |  |  |  |
| Ecig only | 141 | 72.6 | ref |  | 47 | 30.7 | ref |  |
| no meds/ecig/behsup | 295 | 58.8 | 0.54 | (0.33-0.89)* | 528 | 55.6 | 2.83 | (1.38-5.82)** |
| NRT only | 99 | 59.9 | 0.56 | (0.30-1.06) | 260 | 58.5 | 3.18 | (1.50-6.76)** |
| PM only | 73 | 74.9 | 1.12 | (0.53-2.37) | 204 | 67.8 | 4.76 | (2.20-10.30)*** |
| Beh support  Combo/DK/No info | 13  184 | 44.7  75.0 | 0.30  1.13 | (0.07-1.40)  (0.67-1.93) | 27  169 | 65.4  60.3 | 4.27  3.43 | (1.44-12.63)**  (1.57-7.50)** |

Note. N, number of individuals; n, number of observations; AOR, adjusted odds ratios; NRT, nicotine replacement therapy; PM, stop-smoking prescription meds (varenicline or buproprion); LQA, last quit attempt; *p<.05; **p<.01; ***p<.001;

Model adjusted for survey wave, age groups, sex, country, ethnicity, baseline educ, baseline income, baseline HSI, baseline quit intention, baseline recent quit attempt, number of quit attempts, quit recency, survey mode, interwave interval & wave of recruitment; % quit estimates adjusted for covariates in the GEE model;
